# Three-dimensional models of the multiple myeloma bone microenvironment: from disease mechanisms to translational applications

**DOI:** 10.3389/fcell.2026.1954766

**Published:** 2026-10-29

**Authors:** Camilla Sitzia, Cristina Manferdini, Giovanni D’Atri, Gina Lisignoli, Mattia Dessena, Nicola Giuliani, Paola Storti

**Affiliations:** 1 Department of Medicine and Surgery, University of Parma, Parma, Italy; 2 Laboratory of Immunorheumatology and Tissue Regeneration, IRCCS Istituto Ortopedico Rizzoli, Bologna, Italy; 3 Department of Chemistry, Materials and Chemical Engineering “Giulio Natta”, Politecnico di Milano, Milan, Italy; 4 Hematology and BMT Unit, “Azienda Ospedaliero-Universitaria di Parma”, Parma, Italy

**Keywords:** bone microenvironment, multiple myeloma, myeloma bone disease, organ-on-chip, three-dimensional models, tissue engineering

## Abstract

Multiple myeloma (MM) is a hematological malignancy strongly dependent on the bone microenvironment (BME), a dynamic niche that supports malignant plasma cell (PC) survival, immune escape, bone disease, and drug resistance. Conventional two-dimensional (2D) cultures and animal models have provided important insights into MM biology, but they only partially reproduce the spatial organization, cellular complexity, extracellular matrix composition, mechanical cues, and soluble gradients of the human bone marrow (BM). Three-dimensional (3D) models have therefore emerged as complementary experimental platforms to investigate microenvironment-dependent mechanisms of disease progression, therapeutic resistance, and patient-specific drug response. In this review, we provide a critical assessment of both bone-specific and BM like 3D models of MM, with a particular focus on their ability to recapitulate key functional aspects of tumor-bone microenvironment interactions and myeloma bone disease. We differentiate systems that mainly mimic the general BM niche from those that incorporate bone-relevant features, and we evaluate the available platforms based on the biological mechanisms they reproduce, the functional readouts they enable, and their suitability for distinct experimental and therapeutic purposes. We also highlight their relevance for therapeutic target validation and drug testing using patient samples. Finally, we discuss how the integration of patient-derived cells, spatial technologies, and artificial intelligence (AI) may enhance the interpretability and translational value of 3D MM models, ultimately supporting the development of more microenvironment-driven therapeutic approaches.

## 1 Introduction

Multiple myeloma (MM) is a hematologic malignancy characterized by the clonal expansion of antibody-secreting plasma cells (PCs) in the bone marrow (BM) (Kumar et al., 2017) that remains largely incurable, as most patients ultimately relapse or develop refractory disease (Rafae et al., 2024). Progression from premalignant stages, including monoclonal gammopathy of undetermined significance (MGUS) and smoldering multiple myeloma (SMM), to active MM is driven not only by PC-intrinsic genetic and epigenetic lesions, but also by progressive remodeling of the bone microenvironment (BME). The BME is not a passive scaffold, but a dynamic and continuously remodeled compartment in which osteoblasts, osteoclasts, osteocytes, osteogenic progenitors, and the mineralized extracellular matrix (ECM) interact with malignant PCs. These reciprocal interactions disrupt bone homeostasis, promote myeloma bone disease, support tumor progression, and contribute to therapeutic resistance (Iannozzi et al., 2022; Maiso et al., 2021). Understanding how this niche shapes tumor behavior is therefore central to identifying targetable dependencies arising from tumor microenvironment interactions.

Conventional preclinical models have been essential for studying MM biology, yet each carries limitations that constrain their ability to reproduce microenvironment dependent mechanisms. Two-dimensional (2D) cultures offer experimental simplicity but exclude the structural and multicellular complexity of the BM niche, while animal models are constrained by species-specific differences in stromal, immune, and skeletal biology (Bottaro et al., 2025; Ybarra et al., 2025). These limitations are especially relevant for studying microenvironment-mediated drug resistance, which depends on spatial organization and reciprocal tumor niche signaling. Three-dimensional (3D) models have therefore emerged as promising tools to bridge the gap between oversimplified *in vitro* assays and *in vivo* approaches. By capturing essential structural, cellular, and biochemical features of the BME, these models provide mechanistically informative BM like platforms to investigate MM biology, dissect microenvironment-mediated drug resistance, and uncover mechanisms of disease progression and treatment failure (De La Puente et al., 2015; Reagan et al., 2014).

Previous reviews have provided valuable overviews of 3D models for MM, including scaffold-based and scaffold-free systems, patient-derived cultures, and advanced BM-mimetic platforms (Bottaro et al., 2025; Papadimitriou et al., 2020; Verbruggen et al., 2024). However, the ability of these models to reproduce the bone component of the MM microenvironment and the functional alterations underlying myeloma bone disease has been less systematically evaluated. In addition, available 3D platforms are often described according to their technological characteristics or increasing complexity, whereas their suitability for addressing specific biological and therapeutic questions is not always directly compared. In this review, we therefore provide a critical and mechanism-oriented assessment of both bone-specific and BM like 3D models of MM. We distinguish models that primarily reproduce the general BM niche from those incorporating functional bone like features and evaluate their ability to recapitulate defined mechanisms of MM-microenvironment crosstalk, including stromal adhesion, pathological bone remodeling, hypoxia, immune interactions, and microenvironment-mediated therapeutic resistance. We compare the available 3D platforms based on the biological features they reproduce, their cellular and matrix composition, the functional readouts they enable, their reproducibility and scalability, and their suitability for different experimental and therapeutic applications. We further discuss their use for therapeutic target validation and patient-derived drug testing, as well as the potential contribution of spatial technologies and artificial intelligence (AI) to their future development and translational application.

Relevant literature was identified through targeted PubMed searches using combinations of terms related to MM and 3D modeling, including BME, bone remodeling, drug resistance, bioprinting, microfluidics, organ-on-chip systems, and artificial intelligence. Studies published up to August 2026 were considered, with priority given to original articles addressing the key topics discussed in this review.

## 2 The multiple myeloma bone microenvironment: key features for 3D modeling

The BME is a specialized and dynamic component of the BM niche, in which malignant PCs disrupt the balance between bone formation and resorption, promoting osteolytic disease and generating bone-derived signals that support tumor progression and therapeutic resistance (Hideshima et al., 2007; de Jong et al., 2021). Importantly, pathological bone remodeling does not occur in isolation but is influenced by stromal, vascular, immune, hypoxic, and metabolic signals arising from the surrounding BM microenvironment. As the disease progresses, the coordinated remodeling of the bone and marrow compartments enhances osteoclast activation, suppresses osteoblast function, promotes angiogenesis, and reinforces microenvironment-mediated drug resistance (García-Sánchez et al., 2023). Defining the cellular and molecular mechanisms by which the BME sustains MM is therefore crucial for identifying novel therapeutic targets and developing more representative preclinical models. In this section, we focus specifically on the bone-related components of the BME that are most relevant for the development and interpretation of 3D bone models, rather than providing an exhaustive overview of the entire BM microenvironment. Vascular, hypoxic, and other marrow-derived aspects are discussed primarily in relation to their effects on bone remodeling and in the context of advanced 3D and microfluidic models.

### 2.1 Mesenchymal stromal cells and extracellular matrix

Mesenchymal stromal cells (MSCs) are non-hematopoietic cells involved in hematopoietic support, tissue organization, immune regulation, and bone homeostasis (Iannozzi et al., 2022). In MM, malignant PCs progressively reprogram MSCs through reciprocal tumor-stroma crosstalk, leading to altered cytokine secretion, reduced osteogenic potential, and increased angiogenic activity. At the molecular level, MM-reprogrammed MSCs also acquire senescence-associated features and broad transcriptional and epigenetic changes, including miRNA dysregulation (Corre et al., 2007; Plakhova et al., 2025; Garcia-Gomez et al., 2021; Schinke et al., 2018). Early studies showed that BM-MSCs from MM patients display abnormal gene expression profiles and functional alterations compared with MSCs from healthy donors (Arnulf et al., 2007; Bogun et al., 2024). Moreover, García-Gómez et al. showed that interactions between MM cells and MSCs induce broad gene-expression changes in stromal cells, involving pathways related to tumor growth, drug resistance, angiogenesis, osteoclast activation, and osteoblast inhibition (Garcia-Gomez et al., 2014). It is also reported that MSC-related transcriptional signatures may predict clinical outcome and progression from SMM to active MM (Schinke et al., 2018). In addition, single-cell RNAseq and spatial approaches have identified inflammatory stromal populations associated with PCs, immune remodeling, and drug resistance (Cenzano et al., 2026; de Jong et al., 2021). Together, these findings suggest that MM-MSCs acquire a stable disease-associated phenotype that contributes to the formation of a tumor-supportive niche.

The crosstalk between MM cells and MSCs is mediated by direct adhesion, soluble factors, extracellular vesicles, and ECM components (García-Sánchez et al., 2023). Adhesive interactions, mediated by VLA-4/VCAM-1, LFA-1/ICAM-1, CD44-hyaluronan, and integrin-ECM binding, promote PC retention within the BM, activate pro-survival signaling, and contribute to cell adhesion-mediated drug resistance (CAM-DR) (Damiano et al., 1999; Landowski et al., 2003). In parallel, MSC-derived soluble factors, including IL-6, CXCL12/SDF-1α, BAFF, APRIL, IGF-1, HGF, VEGF, and TGF-β, activate key signaling pathways such as JAK/STAT3, PI3K/AKT, MAPK/ERK, and NF-κB. Through these cascades, MSCs sustain PC survival, proliferation, homing, and immune escape, while also promoting angiogenesis and drug resistance (Hideshima et al., 2007; García-Sánchez et al., 2023).

Beyond its structural role, the ECM actively shapes the MM niche through its composition, stiffness, and spatial organization, regulating cell adhesion, migration, mechanotransduction, and drug diffusion (Belloni et al., 2018; Alhallak et al., 2021). Thus, MSC-ECM interactions are central determinants of MM survival and treatment response and provide a functional connection between tumor-supportive stromal signaling and altered skeletal homeostasis. 3D models incorporating MSCs, ECM components, and soluble gradients are therefore particularly relevant, as they can reproduce stromal-dependent mechanisms of progression, osteogenic dysfunction, and therapeutic resistance that remain undetected in conventional 2D cultures.

### 2.2 Osteoblasts, osteoclasts and pathological bone remodeling

Bone remodeling is a tightly coordinated process in which osteoblast (OB)-mediated bone formation and osteoclast (OC)-mediated bone resorption are physiologically coupled to preserve skeletal homeostasis (Roodman, 2009). In MM, disruption of this balance results in pathological uncoupling of bone remodeling, characterized by increased OC activity and impaired OB function, leading to osteolytic lesions and skeletal complications (Lungu et al., 2025; Reagan et al., 2015). Importantly, bone disease actively contributes to MM progression by generating a permissive BME that supports malignant PC survival and growth, rather than representing only a consequence of tumor expansion (Terpos et al., 2019).

Enhanced osteoclastogenesis is driven by tumor- and microenvironment-derived mediators, including RANKL, CCL3/MIP-1α, IL-6, IL-17, TNF-α, and Activin A. Among these, the RANKL/osteoprotegerin imbalance plays a central role, promoting OC differentiation, activation, and bone resorption (Terpos et al., 2018). In parallel, malignant PCs suppress OB differentiation by MSCs through the direct cell-to-cell contact interfering with the osteogenic transcriptional programs, including RUNX2-dependent differentiation and Wnt signaling. High levels of Wnt signaling antagonists such as DKK1 and sclerostin, have been shown in the BME (Terpos et al., 2019; Terpos et al., 2018). This impaired OB activity limits bone repair and contributes to the persistence of osteolytic lesions, which often fail to heal even after tumor burden is reduced (Terpos et al., 2019). In addition, osteocytes, the terminally differentiated cells embedded within the mineralized bone matrix, are increasingly recognized as active contributors to MM bone disease. Recent work has shown that MM-derived CCL3 can induce RANKL expression in osteocytes through a CCL3-HMGB1 signaling axis, thereby promoting bone loss (Anloague et al., 2024).

Emerging evidence also suggests that metabolic alterations within the BM microenvironment contribute to the uncoupling of bone remodeling in MM. High glutamate levels in BM samples from MM patients have recently been associated with increased OC formation and bone resorption, suggesting glutamate transport as a potential target for osteolytic bone disease (Toscani et al., 2025).

Overall, the persistent uncoupling between excessive bone resorption and defective bone formation that characterizes myeloma bone disease is poorly reproduced in conventional 2D cultures. Despite the central role of bone remodeling in MM pathophysiology, the bone compartment remains relatively underexplored in current 3D MM models, which more often reproduce interactions within the BM microenvironment. This gap highlights the need for more representative bone like 3D platforms that integrate functional osteogenic and osteoclastic components with mineralized matrices, enabling quantitative assessment of bone formation and resorption.

## 3 Limitations of conventional preclinical models

Conventional preclinical models have been essential for studying MM microenvironment, identifying disease-associated pathways, and testing therapeutic agents. 2D cultures and animal models remain widely used because they are experimentally accessible, relatively standardized, and suitable for mechanistic studies or *in vivo* validation (Bottaro et al., 2025; Ybarra et al., 2025). However, the strong dependence of MM cells on the BME makes these systems insufficient to fully recapitulate the stromal, immune, and bone-related signals that regulate disease progression and therapeutic response (Bottaro et al., 2025; Ybarra et al., 2025). Although conventional models remain valuable, their translational relevance is limited when studying microenvironment dependent mechanisms, underscoring the need for more physiologically relevant human-based platforms.

### 3.1 Two-dimensional cultures

2D cultures represent the most widely used *in vitro* approach in MM research. Monocultures of MM cell lines (i.e., RPMI-8226, U-266, MM1.S and OPM-2), and less frequently short-term cultures of primary malignant PCs, are inexpensive, reproducible, and compatible with high-throughput drug screening (Bottaro et al., 2025; Ybarra et al., 2025). These systems are useful for studying MM cell-intrinsic pathways, evaluating cytotoxic effects of anti-myeloma agents, and performing genetic or pharmacological perturbations under controlled conditions. However, monocultures largely exclude the BME, which is essential for malignant PC survival, homing, drug resistance, and disease progression (De La Puente et al., 2015).

To partially address this limitation, 2D co-culture systems have been used to expose MM cells to BM-MSCs, endothelial cells, OB like cells, or ECM proteins (Bottaro et al., 2025). These models have shown that stromal contact and soluble factors can protect MM cells from therapy-induced apoptosis and promote microenvironment-mediated resistance (Binder et al., 2024). Mechanistically, BM-MSC-mediated protection has been linked to the upregulation of anti-apoptotic proteins, including MCL-1 and BCL-XL, which cooperate to confer resistance to proteasome inhibitors such as carfilzomib (Galas-Filipowicz et al., 2024). More recent studies have further indicated that PC-MSC interactions induce broad transcriptional and epigenetic changes in MM cells, including chromatin remodeling associated with therapeutic resistance and extramedullary dissemination (Binder et al., 2024). In particular, BM-MSCs can remodel cis-regulatory elements and alter the expression of genes involved in migration and cytokine signaling, with direct implications for patient outcome (Binder et al., 2024). Transwell systems provide a useful approach to distinguish contact-dependent effects from soluble factor-mediated signaling (Dziadowicz et al., 2022). Using this approach, Dziadowicz et al. showed that MSC-derived soluble signals induce transcriptomic and gene-regulatory changes in MM cells that are distinct from monoculture and enriched in JAK2/STAT3 signaling, unfolded protein stress, PC differentiation, and response to proteasome inhibitors (Dziadowicz et al., 2022).

Nevertheless, these systems remain reductionist. They fail to reproduce the BM architecture, physiological ECM properties, mineralized bone matrix, oxygen and nutrient gradients, and the spatial organization of stromal, bone, vascular, and immune compartments. As a consequence, they may overestimate drug sensitivity and systematically underestimate microenvironment-mediated resistance (De La Puente et al., 2015; Belloni et al., 2018). Therefore, 2D systems remain useful for rapid screening and mechanistic studies, but their inability to reproduce the spatial and multicellular complexity of the BM niche supports the use of 3D models as complementary platforms (Ybarra et al., 2025; Alhallak et al., 2021).

### 3.2 Murine models

Animal models of MM span several experimental systems designed to reproduce selected aspects of disease biology, each with specific strengths and constraints. Syngeneic immunocompetent models (primarily the 5TMM series) support tumor growth in the presence of an intact murine immune system and have been valuable for studying bone disease, angiogenesis, and immune regulation (Vanderkerken et al., 2005; Mehdi et al., 2021). Among these, the 5T2MM model has been used to evaluate tumor burden, serum paraprotein, plasmacytosis, bone lesions, and angiogenesis (Vanderkerken et al., 2005). The 5TGM1 model is widely used for myeloma bone disease because it reproduces BM localization, monoclonal protein production, osteolytic lesions, and altered bone remodeling (Mehdi et al., 2021). Luciferase-expressing 5TGM1 models have improved longitudinal monitoring of tumor burden and skeletal lesions, supporting more standardized evaluation of disease progression and bone-targeted therapies (Mehdi et al., 2021). Recent 5TGM1 transfer studies have also highlighted the contribution of host immunity, showing that stable disease is associated with sustained activation of NK cells, Type I innate lymphoid cells (ILC1s), and CD8^+^ T cells and with IFN-γ-mediated tumor control (Kellermayer et al., 2024).

Xenograft models based on human MM cell lines or primary MM cells allow the *in vivo* assessment of human tumor growth and drug activity. However, their dependence on immunodeficient hosts limits the study of adaptive immunity, immune escape, and immunotherapy response (Yata et al., 2004). Subcutaneous xenografts are experimentally accessible but poorly reproduce BM localization, whereas orthotopic or intravenous models better approximate BM homing while still relying on murine stromal and skeletal compartments (Ybarra et al., 2025; Yata et al., 2004). Humanized bone models, including SCID-hu, SCID-rab, and SCID-synth-hu systems, better support MM growth within a human bone compartment and are useful for studying primary PC engraftment, osteolysis, and tumor-bone interactions (Yata et al., 2004; Calimeri et al., 2011). Nevertheless, these platforms remain technically demanding, expensive, low-throughput, and dependent on immunodeficient hosts.

Genetically engineered and humanized mouse models have expanded the study of MM initiation, clonal evolution, and immune surveillance in immunocompetent settings. Humanized mouse models have supported the BM-restricted engraftment and growth of primary human preneoplastic and malignant PCs while preserving key genomic features of the corresponding patient samples, thereby highlighting the dependence of tumor cells on microenvironmental support (Das et al., 2016). Moreover, recent genomic profiling showed that the Vk*MYC model shares transformation pathways with human MM, supporting its value for studying disease evolution (Maura et al., 2024). In addition, genetically heterogeneous MM mouse models have linked recurrent MM-associated lesions with immune evasion and immunotherapy response, including resistance to immune checkpoint blockade (Larrayoz et al., 2023). Overall, no animal model fully recapitulates the human MM BME, and each system should be selected based on the specific biological question being addressed. Human-based 3D systems are therefore needed as complementary platforms to study tumor-microenvironment interactions in a more controlled and patient-relevant setting (Ybarra et al., 2025; Larrayoz et al., 2023).

## 4 Three-dimensional models of the multiple myeloma bone marrow microenvironment

The principal 3D modeling strategies currently employed to reproduce the MM BM microenvironment are summarized in Figure 1, highlighting the progression from scaffold-free spheroids to advanced tissue engineered, bioprinted, and organ-on-chip models.

**FIGURE 1:**
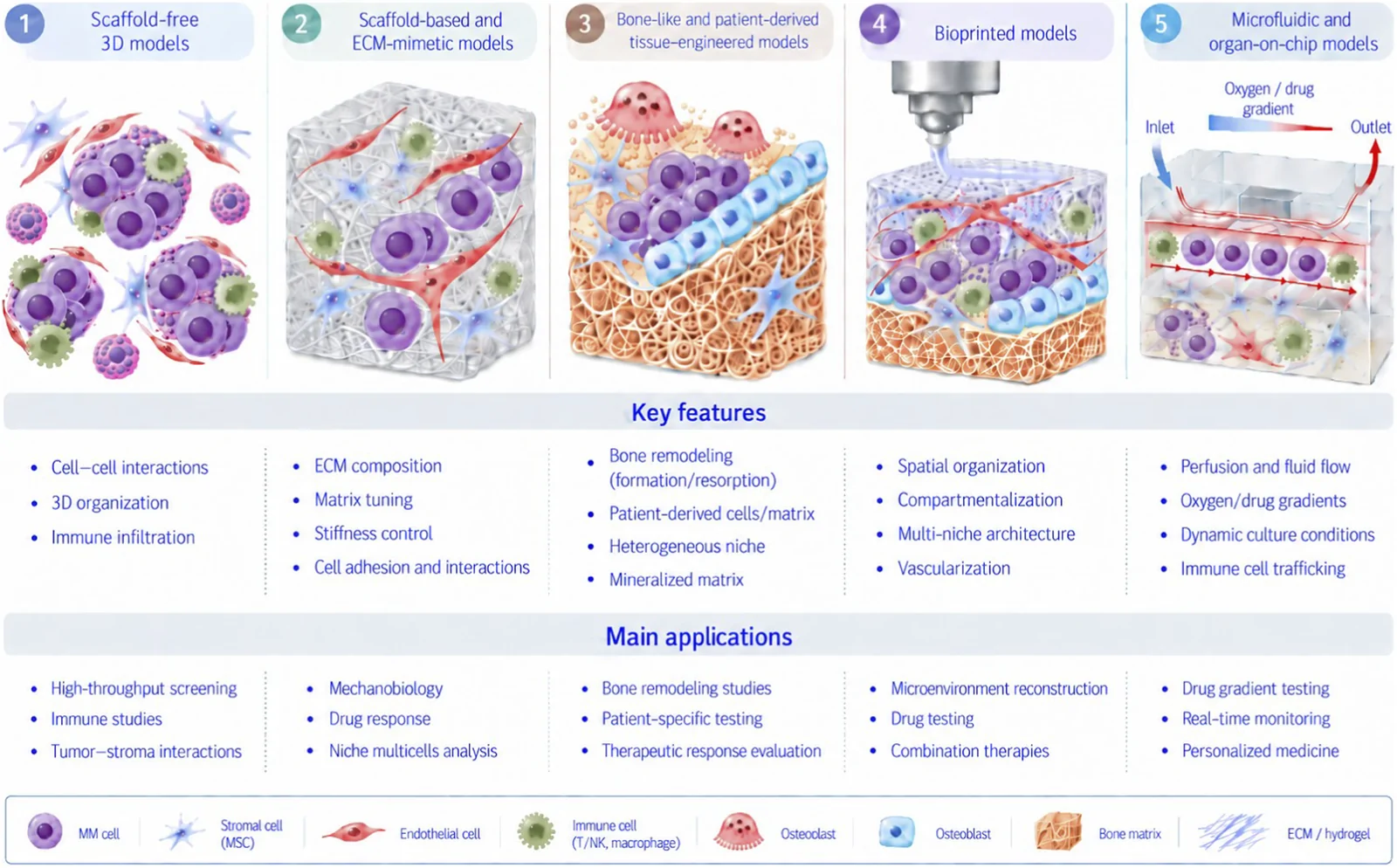
Schematic overview of the main three-dimensional (3D) models used to reproduce the multiple myeloma (MM) bone marrow (BM) microenvironment. The figure summarizes the key features and representative applications of scaffold-free, scaffold-based/ECM-mimetic, bone like and patient-derived tissue-engineered, bioprinted, and microfluidic/organ-on-chip models. Each platform offers distinct advantages depending on the biological question and required readouts, and increasing model complexity does not necessarily correspond to greater physiological relevance.

### 4.1 Scaffold-free 3D models: spheroids, organoids and multicellular aggregates

Scaffold-free 3D systems include spheroids, organoids, and multicellular aggregates, which differ in their degree of cellular organization and functional complexity. Spheroids are self-assembled aggregates generated from one or more defined cell populations, whereas organoids are self-organizing structures that reproduce selected cellular and functional features of the tissue of origin. Multicellular aggregates represent a broader category of heterogeneous 3D cell assemblies that do not necessarily exhibit organoid like organization or function (Bottaro et al., 2025; Verbruggen et al., 2024).

To reproduce selected features of the MM BM niche, scaffold-free 3D models have been developed as powerful platforms for both basic research and drug-response evaluation. Different MM cell lines, including U-266, AMO-1, NCI-H929, and SKMM1, as well as primary PCs, have been used either alone or in co-culture with MSCs, endothelial cells, or immune effector cells to investigate interactions among representative niche cell types and their contribution to drug resistance. Waldschmidt et al. developed a novel high-throughput 3D spheroid co-culture system using primary MM cells in monoculture or in co-culture with MSCs, which supported sustained cell proliferation for up to 21 days (Waldschmidt et al., 2022). They showed that bortezomib and auranofin induced lower cytotoxicity in this 3D co-culture system than in 3D monoculture or 2D conditions (Waldschmidt et al., 2022). To recapitulate tumor-stroma interactions in MM and study CAR-immune cell-mediated cytotoxicity, Luanpitpong et al. developed a high-throughput 3D model (Luanpitpong et al., 2025). They demonstrated that cancer-associated fibroblast-like stromal cells co-cultured with MM cells provided a more comprehensive immune oncology model of tumor-stroma interactions and improved the ability of NK cells to infiltrate 3D spheroids (Luanpitpong et al., 2025).

Shen et al. combined human bone and lympho-myeloid BM to generate a preclinical organoid model, termed combined bone and lympho-myeloid organoids (comBOs), and demonstrated that this system supported the engraftment and survival of primary MM cells *ex vivo* for up to 2 weeks (Shen et al., 2026). Single-cell RNA sequencing showed that most MM cells were in G1, with a minority in G2/M and S phases, confirming the low proliferative rate characteristic of myeloma. These cells also expressed canonical myeloma markers, including BCL2, IRF4, and SLAMF7. Moreover, gene-set enrichment analysis identified hallmark gene sets that captured myeloma-associated multilineage and microenvironmental remodeling within comBOs (Shen et al., 2026).

### 4.2 Scaffold-based and ECM-mimetic models

To recreate the ECM compartment, two main classes of scaffolds have been used in MM culture models: scaffolds-derived from natural or synthetic polymers. These scaffolds have been used to seed or embed isolated MM cells, including cell lines and patient-derived cells, as well as cell spheroids or organoids. Hydrogels provide a supportive environment for MM cells and are useful for drug-response and immune-related studies, whereas spheroids and organoids help simulate tissue-like clusters that better reproduce native tumor architecture and immune responses.

Scaffolds derived from natural polymers (i.e. hyaluronic acid, gelatin, chitosan, collagen, alginate) or synthetic polymers (i.e. PLA, PEG, PCL) are widely used in MM culture models, mainly as hydrogels or bioinks. Among natural polymers, Narayanan et al. developed a hyaluronic acid-based hydrogel to recapitulate physiological conditions of the MM cell microenvironment (Narayanan et al., 2014). This model supported MM cell survival and migration and induced the expression of adhesion-related proteins involved in the Wnt/β-catenin signaling pathway, which may influence drug response (Narayanan et al., 2014). To investigate MM pathogenesis and drug response, Belloni et al. developed a 3D rotary cell culture system based on gelatin scaffolds that supported the co-culture of MM cells with BM-derived stromal components. This model reproduced tumor-supportive microenvironmental interactions and cell adhesion-mediated drug resistance (Belloni et al., 2018). They also applied this system to co-culture primary MM cells with MSCs and endothelial cells, showing that it favored functional myeloma-stroma interactions, long-term MM cell survival, and evaluation of the effects of bortezomib on MM cells (Belloni et al., 2018). Other studies have used microgel systems functionalized with hyaluronic acid, collagen, dextran, or alginate to support MM cell proliferation and resistance to anti-MM drugs (Clara-Trujillo et al., 2022), reproduce interactions between tumor PCs and their BME (García-Briega et al., 2025), or support monoclonal PCs as a disease model for MM (Marín-Payá et al., 2021).

Commercial natural hydrogel-based scaffolds have also been widely used. Among them, Matrigel, either alone or in combination with fibrinogen or collagen, has been extensively employed (Khan et al., 2023). Kirshner et al. developed a Matrigel-based 3D culture model to support MM cells within a reconstructed *in vitro* microenvironment (Kirshner et al., 2008). Under these conditions, MM clones expanded up to 17-fold, including malignant cells harboring clonotypic IgH VDJ rearrangements (Kirshner et al., 2008). Moreover, this model showed that melphalan mainly targeted hematopoietic cells, whereas bortezomib selectively targeted CD138^+^CD56^+^ MM PCs, providing a relevant preclinical model to assess the vulnerability of the whole MM clone, including drug-resistant CD20^+^ MM subpopulations (Kirshner et al., 2008). Cucè et al. developed a Matrigel-based 3D model by embedding MM cell lines or patient-derived MM cells either alone or in combination with human monocytes and tested the effects of trabectedin (Cucè et al., 2019). In this model, trabectedin induced apoptosis, DNA damage response activation, ROS production, and cell-cycle arrest, leading to upregulation of NK-activating receptors (Cucè et al., 2019). More recently, Lourenço et al. developed a 3D *ex vivo* BM culture system by embedding unmanipulated MM BM aspirates in Matrigel (Lourenço et al., 2026). This system maintained the viability of hematopoietic, stromal, and immune cells for up to 7 days, preserved key cytokines of the MM microenvironment, and allowed different cell populations to exhibit spatial organization and interactions. It also supported drug-sensitivity testing, enabling discrimination of treatment responses in MM cell lines (Lourenço et al., 2026).

Among synthetic polymers, Spelat et al. developed a worm gel-based 3D model to mimic paracrine interactions between MM cells and MSCs, identifying synergistic IL-6/IL-10 signaling as a critical pathway supporting MM proliferation (Spelat et al., 2020). Sprules et al. developed a 3D polycaprolactone-based polymerized High Internal Phase Emulsion (polyHIPE) scaffold model that mimics the osteoblastic bone niche, where myeloma dormancy may occur (Sprules et al., 2026). Using different MM cell lines, including U-266, JJN3, and 5TGM1, they demonstrated that polyHIPE scaffolds facilitated OB and MM cell infiltration and interactions, while also altering cellular sensitivity to melphalan, bortezomib, and lenalidomide (Sprules et al., 2026). Jakubikova et al. developed a 3D MM model using the synthetic PuraMatrix™ hydrogel, in which BM mononuclear cells from MM patients were co-cultured with autologous or allogeneic MSCs (Jakubikova et al., 2016). This model increased MM cell proliferation and induced the production of MSC-derived cytokines, including IL-6 and IL-8, as well as ECM and adhesion molecules, including VLA-4, VLA-2, and VLA-5, which are involved in MM cell proliferation and stromal interactions. Moreover, the model reproduced resistance to both conventional and novel drugs, including bortezomib and doxorubicin, reflecting clinical resistance patterns (Jakubikova et al., 2016).

Scaffold stiffness has recently emerged as an important parameter for reproducing the complexity of the tumor microenvironment, as solid tumors are often stiffer than their healthy counterparts (Bhatt et al., 2026). This biophysical cue may influence tumor cell survival, invasiveness, and resistance to therapy. Although stiffness has been extensively studied in solid tumors (Deng et al., 2022) and bone tissue stiffness is well documented (Piersma et al., 2020), its role in the BM niche remains poorly explored in MM. Therefore, tuning scaffold stiffness may represent an important strategy for developing more physiologically relevant *in vitro* 3D MM models and for investigating how biomechanical cues influence MM cell behavior and therapeutic response.

### 4.3 Bone like and patient-derived tissue-engineered bone marrow models

BM like models reproduce selected cellular, extracellular, and functional features of the BM microenvironment, whereas bone like models additionally incorporate bone-specific features, including mineralized matrix, osteogenic maturation, OC activity, or evidence of bone remodeling. The presence of bone-associated cells alone is not sufficient to classify a model as bone like. Models combining BM and bone-specific features are referred to as multiniche BM/bone models.

Advanced bone like tissue-engineered models have been developed to reproduce biochemical, structural, and mechanical features of the MM BM niche. By integrating mineralized matrices with OBs, OCs, MSCs, endothelial cells, and ECM components, these systems recreate the complex cellular crosstalk that drives MM progression, bone disease, and therapeutic resistance (Reagan and Ghobrial, 2012; Fairfield et al., 2016). De la Puente et al. developed a 3D tissue-engineered BM model that combined MM cells with a reconstructed BME to reproduce key pathological features of the disease, including enhanced tumor growth, cell-cell interactions, and microenvironment-mediated drug resistance (De La Puente et al., 2015). Compared with conventional 2D cultures, tissue-engineered BM models provide a more BM like and mechanistically informative model for studying MM biology and evaluating therapeutic responses.

Decellularized bone and BM matrices represent another promising approach, as they preserve native ECM composition, tissue-specific ligand presentation, and biomechanical cues. By maintaining the complexity of the original microenvironment, these systems may better support the survival, phenotype, and functional behavior of primary MM cells than conventional biomaterials. However, the decellularization process requires specific technologies and methods for sterilization that limit the reproducibility and tunability, mainly because of the process needed to remove cellular components from animal tissues (Kasravi et al., 2023). Qi et al. used decellularized extracellular matrix (dECM) of normal bone to evaluate MM cell viability and sensitivity to bortezomib, showing that dECM may inhibit MM development through the APRIL-TACI axis (Qi et al., 2021).

Patient-derived tissue-engineered models further extend this concept by incorporating primary MM cells together with autologous stromal and immune components. Such models preserve patient-specific cellular heterogeneity and drug response profiles, making them particularly attractive for precision medicine applications. For example, MSC-based 3D co-culture systems and *ex vivo* BM models have demonstrated improved maintenance of primary MM cells and enhanced predictive value for therapeutic testing compared with standard *in vitro* cultures (Jakubikova et al., 2016). Notably, Alhallak et al. developed a novel patient-derived model to retrospectively predict clinical responses to different therapeutic regimens (Alhallak et al., 2021). The model incorporated accessory and primary cancer cells present in the BM within a fibrinogen-based matrix, which was cross-linked with calcium and tranexamic acid to generate a hydrogel-like 3D structure mimicking the BM niche. Notably, the authors demonstrated a high correlation between this system and clinical responses in patients, retrospectively predicting clinical responses to different treatment regimens, including bortezomib, doxorubicin, and carfilzomib, in 89% of the MM cohort (Alhallak et al., 2021).

Together, bone like and patient-derived tissue-engineered models bridge the gap between simplified *in vitro* systems and the complexity of the native BM niche, providing powerful tools for investigating disease mechanisms, microenvironment-driven resistance, and patient-specific therapeutic responses (Belloni et al., 2018).

### 4.4 Bioprinted models

3D bioprinting has emerged as a powerful strategy for recreating the spatial complexity of the MM BM niche. In contrast to conventional scaffold-based systems, bioprinting allows precise control of cellular distribution, matrix composition, and tissue architecture by depositing bioinks containing living cells and biomaterials in layers (Murphy and Atala, 2014). This approach enables the generation of compartmentalized microenvironments that mimic key BM niches, including endosteal and perivascular regions, providing a valuable platform for studying PC homing, disease progression, and microenvironment-mediated drug resistance (De La Puente et al., 2015).

One of the earliest applications of bioprinting in MM was reported by Braham et al., who developed a 3D BM model integrating bioprinted calcium phosphate cement (CPC) scaffolds to mimic the mineralized endosteal niche, together with a Matrigel-based perivascular compartment containing MSCs and endothelial progenitor cells (Braham et al., 2018). This multicompartment system supported the survival and localization of primary CD138^+^ PCs and highlighted the distinct contributions of endosteal and perivascular microenvironments to MM cell behavior. Importantly, the study demonstrated the feasibility of combining bioprinted bone like structures with biologically relevant cellular niches to reproduce the complexity of the BM ecosystem *in vitro* (Braham et al., 2018). Although quantitative stiffness measurements were not reported, the use of a mineralized CPC scaffold introduced a mechanically distinct compartment that more closely resembled the rigid endosteal bone surface (Braham et al., 2018).

Beyond architectural complexity, bioprinting offers the possibility of reproducing the mechanical heterogeneity of the BM niche. In MM, however, this aspect remains relatively underexplored, as only a limited number of bioprinted models have reported quantitative mechanical characterization of engineered constructs. Nevertheless, the incorporation of mineralized materials such as CPC and nano-hydroxyapatite (nHA) introduces stiffness contrasts that better mimic the transition from the rigid bone interface to the softer marrow environment. Such biomechanical gradients are increasingly recognized as important regulators of cell adhesion, migration, proliferation, survival, and drug responsiveness within the tumor microenvironment.

Wu et al. developed a coaxially bioprinted BM model composed of an nHA-enriched outer sheath surrounding a hydrogel core containing MM cells and HS-5 stromal cells (Wu et al., 2022). The construct was fabricated using a combination of gelatin methacryloyl (GelMA), alginate, polyethylene glycol diacrylate (PEGDA), and nHA, allowing modulation of both printability and mechanical properties. The hydrogel core was engineered to exhibit relatively low stiffness, within the kPa range, consistent with the compliant nature of the BME. Mechanical characterization by compression testing demonstrated that the incorporation of polymeric and mineral components increased scaffold stiffness while preserving high cell viability and structural integrity. The resulting bone-mimetic architecture promoted MM cell proliferation and aggregation, enhanced IL-6 secretion, and reproduced stromal-mediated resistance to bortezomib more effectively than conventional 2D cultures. Moreover, inhibition of IL-6 signaling with tocilizumab restored sensitivity to bortezomib, highlighting the utility of bioprinted systems for identifying microenvironment dependent therapeutic vulnerabilities. Importantly, the model also prolonged the *ex vivo* survival of patient-derived MM cells, overcoming one of the major limitations of *in vitro* MM cultures (Wu et al., 2022).

Despite the intrinsic potential of bioprinting to precisely tune matrix stiffness and generate mechanically heterogeneous microenvironments, current MM bioprinted models have primarily focused on reproducing tissue architecture, cellular composition, and drug-response profiles. Systematic investigations directly linking scaffold stiffness to MM cell behavior, mechanotransduction pathways, and therapeutic response remain largely absent. Considering the growing evidence that mechanobiological pathways regulate cancer progression and therapeutic response, future bioprinted MM models incorporating controlled stiffness gradients and quantitative mechanical characterization may provide valuable insights into the biomechanical regulation of disease progression and treatment resistance.

Overall, bioprinted MM models represent promising next-generation platforms for reproducing the spatial complexity of the BM niche and for integrating patient-derived material, stromal interactions, and drug-response analyses into highly controllable *in vitro* systems. The evaluation of bioprinted scaffolds that recapitulate the mechanical properties of BM tissue may help elucidate the stiffness-dependent role of the BM in disease progression (Rogkoti et al., 2025). Furthermore, anatomical location and BM remodeling associated with MM progression should also be considered, as they are important factors for accurately reproducing the characteristics of native healthy BM *in vitro* and comparing them with those of BM affected by MM.

### 4.5 Microfluidic and organ-on-chip models

Microfluidic and organ-on-chip technologies represent a major step toward dynamic, perfusable, and compartmentalized modeling of the MM BME. In contrast to static culture systems, these models incorporate controlled fluid flow, nutrient exchange, oxygen gradients, and mechanical stimulation, enabling the recreation of key aspects of BM physiology, including hypoxic conditions, cell trafficking, and microenvironmental remodeling (Bhatia and Ingber, 2014; Azab et al., 2012). Early patient-specific microfluidic systems demonstrated that primary MM cells could be maintained *ex vivo* only in the presence of a reconstructed endosteal niche containing OBs and ECM components, highlighting the critical role of BM-derived survival signals and dynamic perfusion conditions. These models also enabled real-time monitoring of MM cell migration, adhesion, and clonal expansion within a biomimetic 3D environment (Zhang et al., 2014).

More recently, vascularized MM-on-chip models have integrated endosteal and perivascular niches through the incorporation of MSCs, OBs, endothelial cells, and perfusable microvascular networks. These systems recapitulate key ECM components of the BM, including collagen I, fibronectin, osteopontin, collagen IV, and hyaluronan, while supporting the long-term survival of both MM cell lines and patient-derived PCs. Interestingly, MM cell lines preferentially localized within perivascular regions, whereas primary patient cells were distributed across multiple BM compartments, reflecting the biological heterogeneity of the disease (Ghoshal et al., 2025). Beyond disease modeling, organ-on-chip technologies are emerging as valuable tools for immunotherapy research. Perfusable vascular networks allow the dynamic delivery of anti-BCMA CAR-T cells and enable real-time assessment of immune-cell trafficking, cytokine production, proliferation, and tumor cytotoxicity within a vascularized BM like microenvironment (Ghoshal et al., 2025). Sui et al. developed a microfluidic device designed to recapitulate the sinusoidal niche of the BM and investigated the trafficking of MM cells through a vascular like compartment under controlled microfluidic conditions. This approach provides a dynamic model to study tumor cell migration and interactions with the vascular niche and may therefore represent a useful framework for further investigating therapeutic cell trafficking and response, including in the context of CAR-T cell-based therapies (Sui et al., 2022). By integrating vascular, stromal, immune, and tumor compartments within a single platform, MM-on-chip systems provide powerful tools for investigating microenvironment-driven therapeutic resistance, evaluating patient-specific treatment responses, and reducing reliance on animal models. Future developments are expected to combine organ-on-chip technologies with patient-derived tissues, high-content imaging, and computational modeling to create increasingly predictive platforms for precision medicine. The integration of multiple BM niches and dynamic disease states may ultimately enable the study of MM progression from early disease stages within a single experimental framework (Verbruggen et al., 2024).

The development of BM-on-chip models incorporating hydrogels with tunable stiffness, together with controlled mechanical stimulation, could provide new insights into how biomechanical cues influence PC localization, proliferation, and therapy resistance.

### 4.6 Comparative considerations for 3D model selection

The models summarized in Table 1 should be considered complementary models rather than a linear progression in which increasing complexity necessarily results in greater physiological relevance. A key consideration is the balance between biological fidelity and experimental standardization. Simpler scaffold-free and hydrogel-based models generally offer more accessible and controllable experimental workflows, whereas multicellular tissue-engineered, bioprinted, and microfluidic models provide opportunities to reproduce a broader range of microenvironmental features but may require more complex fabrication, culture, or analytical procedures (Belloni et al., 2018; Waldschmidt et al., 2022; Luanpitpong et al., 2025; Shen et al., 2026; Jakubikova et al., 2016; Braham et al., 2018; Wu et al., 2022; Ghoshal et al., 2025; Sui et al., 2022). The use of patient-derived material can increase biological relevance by preserving disease- and patient-specific cellular characteristics. However, these models are intrinsically influenced by the availability and quality of clinical samples and by interpatient heterogeneity, which can affect experimental reproducibility and scalability (Alhallak et al., 2021; Kirshner et al., 2008; Jakubikova et al., 2016). This tradeoff is particularly relevant when models are intended for repeated comparative studies or drug-screening applications, for which standardized cell sources may provide greater experimental consistency. The appropriate model also depends on the experimental endpoint. Models with relatively limited cellular complexity can address endpoints such as MM cell survival, proliferation, and response to anti-myeloma agents (Waldschmidt et al., 2022; Narayanan et al., 2014; Kirshner et al., 2008; Cucè et al., 2019), whereas multicellular and structurally organized models allow investigation of more specific microenvironment dependent processes, including stromal interactions, bone associated responses, immune cell activity, endothelial function, and cell trafficking (Belloni et al., 2018; Sprules et al., 2026; Jakubikova et al., 2016; Braham et al., 2018; Ghoshal et al., 2025; Sui et al., 2022). Patient-derived models may additionally support *ex vivo* therapeutic-response assessment and personalized drug testing, although their translational value remains dependent on further validation in larger and independent cohorts (Alhallak et al., 2021; Jakubikova et al., 2016). Accordingly, model selection should be driven primarily by the biological question, required readout, and practical constraints rather than by overall model complexity. Standardized models may be preferable for reproducible and scalable comparative studies, whereas patient-derived, multicellular, bone-associated, or dynamic microfluidic models may be more appropriate when the objective is to investigate specific microenvironment dependent mechanisms or patient specific therapeutic responses.

**TABLE 1:** Overview of three-dimensional (3D) models of the multiple myeloma (MM) microenvironment. Comparison of scaffold types, cellular composition, experimental applications, bone, immune, and vascular components, advantages, limitations, and key references.

3D model	Scaffold types	Cell types	Applications	Bone/Immune/Vascular component (B/I/V)	Advantages	Limitations	References
Scaffold-free model: Spheroids	None	Primary MM cells; MM cell lines; stromal cell lines	Primary MM *ex vivo* propagation	B: No I: No V: No	Supports *ex vivo* MM expansion; long-term culture; drug response	Limited cellular complexity	Waldschmidt et al. (2022)
Scaffold-free model: Spheroids	None	MM cell lines; mantle cell lymphoma; normal fibroblasts; BM-derived MSC line	TME reconstruction; high-throughput CAR-immune cell screening	B: No I: CAR-immune cells V: No	Enables assessment of CAR-immune cell infiltration in stromal/CAF-like spheroids	High variability in tumor response; incomplete TME-cell representation	Luanpitpong et al. (2025)
Scaffold-free model: Organoids	None	Human iPSC lines; primary MM cells	BM reconstruction	B: Osteolineage cells, OBs, OCs I: lymphoid, myeloid, T, NK cells V: CD34+ cells	Multi-niche organoid recapitulating complex human BM; drug-response testing (immune/vascular compartments)	Vascular perfusion strategies required	Shen et al. (2026)
Scaffold-based model	Natural HA-based hydrogel	MM-MSCs	MM microenvironment modeling	B: No I: No V: No	3D MM model for phenotypic changes and drug response	Limited cellular complexity	Narayanan et al. (2014)
Scaffold-based model	Natural gelatin-based hydrogel	MM cell lines; primary MM cells + MSCs; HUVECs	MM BM co-culture; tumor-stroma interactions; drug response	B: OBs I: No V: HUVEC	Validation with different cell lines; interaction tumor–stroma, evaluation of clone formation and drug response	Single scaffold limits microenvironment complexity	Belloni et al. (2018)
Scaffold-based model	Hybrid acrylic microspheres with FN/HA functionalization	MM cell lines	Semi-solid MM BM microenvironment; suspension culture	B: No I: No V: No	Tunable microsphere size/agitation/functionality; supports proliferation and drug response	Limited cellular complexity	Clara-Trujillo et al. (2022)
Scaffold-based model	Natural HA/CS/HS/Col I-functionalized dextran microspheres	MM cell lines	Semi-solid MM BM microenvironment; MM PC–MSCs interactions; drug screening	B: No I: No V: No	Versatile ECM-functionalized system for MM cells–ECM interactions	Limited cellular complexity	García-Briega et al (2025)
Scaffold-based model	Natural alginate microspheres with HA/Col I functionalization	MM cell line monoclonal plasma cells (mPC)	Semi-solid BM microenvironment; drug screening	B: No I: No V: No	Versatile ECM-functionalized system for mPC–ECM interactions	Limited cellular complexity	Marín-Payá et al. (2021)
Scaffold-based model	Natural collagen I:matrigel (70:30) hydrogel	Vascular/hematopoietic-lineage iPSC line; primary MM CD138+ PC	Crosstalk among hematopoietic, endothelial and stromal cells; pharmacogenomics/drug studies	B: No I: Hematopoietic/myeloid cells V: Endothelial cells	Mimics BM niche features; supports MM engraftment and allows pharmacogenomic screening	Limited cellular complexity; lacks sinusoidal-like endothelium and osteoid lineage	Khan et al. (2023)
Scaffold-based model	Natural matrigel:fibronectin (2:1) hydrogel	Primary BM MM mononuclear cells; MM BM aspirates with bone attached	*Ex vivo* BM microenvironment	B: OBs, OCs I: B/T cells, monocytes V: No	Supports MM BM clone expansion and BM aspirate characterization	Single scaffold limits microenvironment complexity	Kirshner et al. (2008)
Scaffold-based model	Natural matrigel hydrogel	MM cell lines; primary MM CD138+ PC; healthy CD14^+^ monocytes	3D BM architecture; monocyte–MM interactions; drug screening	B: No I: Monocyte V: No	Supports MM viability/proliferation and drug screening	Single scaffold limits microenvironment complexity	Cucè et al. (2019)
Scaffold-based model	Natural matrigel hydrogel	MM cell lines; MM BM aspirates with bone attached	*Ex vivo* BM microenvironment; MM cell/BM aspirate studies	B: No I: Hematopoietic/myeloid cells V: NR	Supports MM viability and drug screening	Single scaffold limits microenvironment complexity	Lourenço et al. (2026)
Scaffold-based model	Synthetic PGMA-PHPMA-based worm gel	MM cell lines; human MSCs	MM–MSC paracrine interaction	B: No I: No V: No	Supports paracrine MM–MSC interaction studies	Single scaffold limits microenvironment complexity	Spelat et al. (2020)
Scaffold-based model	Synthetic methacrylated PCL polyHIPE scaffold	MM cell lines; osteoblastic cell lines	Osteoblastic bone niche; cell infiltration; MM dormancy/drug response	B: OBs I: No V: No	Bone-niche interaction	Specialized equipment required to develop scaffold	Sprules et al. (2026)
Scaffold-based model	Synthetic PuraMatrix™ peptide hydrogel	Autologous/allogeneic BM primary MM cells (MSCs and mononuclear cells); MM cell lines	MM niche modeling; pathogenesis; drug resistance	B: NR I: B, T, NK cells V: No	Allow studies of MM pathogenesis and drug resistance	Single scaffold limits microenvironment complexity	Jakubikova et al. (2016)
Patient-derived scaffold- based model	3DTEBM crosslinked with endogenous fibrinogen	MM cell lines; Primary BM samples from MM patients	BM tissue niche modeling and drug response prediction	B: No I: No V: Endothelial cells	Patient-derived; reproduces BM TME; oxygen gradients; clinically actionable; 89% retrospective prediction	Pilot study; requires prospective validation	Alhallak et al. (2021)
Patient-derived scaffold-based model	3DTEBM crosslinked with endogenous fibrinogen; Autologous BM supernatant	Primary MM cells + BM accessory cells; MM lines; stromal/endothelial cells	Personalized drug screening; prediction of therapeutic response; MM biology	B: No I: No V: Endothelial cells	Highly patient-specific; physiological BM milieu; rapid; low exogenous material	Requires fresh BM aspirates; lacks blood-flow/shear; limited metastasis modeling	De La Puente et al. (2015)
Bone-derived dECM 3D model	Decellularized rat skull ECM	H929, U-266, RPMI-8226; HS-5	MM–ECM interactions; bortezomib response	B: Bone-derived dECM I: No V: No	Bone-derived ECM; simple preparation; enhanced drug sensitivity	Non-human ECM; lacks immune and vascular compartments	Qi et al. (2021)
Patient-derived scaffold-based model	Synthetic PuraMatrix™ peptide hydrogel	MSCs; primary MM cells; MM lines; HSCs; immune cells; endothelial cells	MM pathogenesis; drug resistance; personalized drug testing; immune profiling	B: NR I: PCs, NK, B-T cells V: No	Patient-derived; immune complexity; models drug resistance	No vascular niche; incomplete BM architecture; prospective validation required	Jakubikova et al. (2016)
Patient-derived scaffold-based model	Natural gelatin-based hydrogel	Primary MM cells; MSCs; accessory BM cells; MM lines	Personalized drug response; *ex vivo* therapeutic prediction	B: OBs I: No V: HUVEC	Highly physiological; rapid; patient-specific; predictive potential	Requires patient samples; no perfusion; limited prospective validation	Belloni et al. (2018)
3D bioprinted model	Calcium phosphate cement + Matrigel	OPM-2, L363; MSCs/O-MSCs; primary CD138+ MM cells; EPCs	MM–bone interactions; osteolysis; drug response	B: CPC/osteogenic compartment I: No V: EPCs/endothelial progenitor cells	Dual endosteal/perivascular niches; primary MM support; spatial organization	Lacks immune/hematopoietic component; imaging limitations; technical complexity	Braham et al. (2018)
3D bioprinted model	GelMA/alginate/PEGDA/nHA bioink	MM1.S, RPMI-8226; HS-5; primary MM cells	MM progression; drug resistance; personalized therapy; high-content screening	B: Mineralized/nHA I: No V: No	Spatially organized; bone-mimetic; supports primary MM cells; tunable mechanics	Limited immune/vascular components; bioprinting-related anisotropy/geometry	Wu et al. (2022)
Microvascularized BM-on-a-chip model	Fibrin/collagen I hydrogel; ECM-based niches	MSCs, HUVECs, fibroblasts; MM lines; primary BM cells; T/CAR-T cells	MM–stroma interactions; CAR-T trafficking/response; therapeutic testing	B: OBs, OCs I: NK, B, T cells V: Endothelial cells	Multiniche BM architecture; perfusion; patient-derived cells; immune–tumor interactions	Technically complex; imaging/quantification constraints	Ghoshal et al. (2025)
Microfluidic ossified BM-on-a-chip model	Osteoblast-derived ECM; PCL mesh	hFOB 1.19; primary MM cells; BMMCs	Personalized drug testing; MM–bone interactions; drug resistance	B: OB/ossified tissue I: No V: No	Small sample requirement; dynamic microenvironment; patient-specific testing	Loss of non-adherent cells; lacks immune/vascular complexity	Zhang et al. (2014)
Microfluidic ossified BM-on-a-chip model	Type I rat tail collagen (5153, Advanced BioMatrix)	MM.1S + HS-5 MSCs + EA.hy926 ECs	MM cell trafficking; CXCL12-mediated egression; endothelial barrier function	B: No I: No V: Endothelial	Dynamic sinusoidal flow; stromal vascular compartmentalization; trafficking studies	Cell lines only; no osteoblasts; limited BM complexity	Sui et al. (2022)

Abbreviations: 3DTEBM, three-dimensional tissue-engineered bone marrow; BM, bone marrow; BMMC, bone marrow mononuclear cell; CAF, cancer-associated fibroblast; CAR, chimeric antigen receptor; CAR-T, chimeric antigen receptor T cell; Col I, collagen type I; CPC, calcium phosphate cement; CS, chondroitin sulfate; CXCL12, C-X-C motif chemokine ligand 12; dECM, decellularized extracellular matrix; EC, endothelial cell; ECM, extracellular matrix; EPC, endothelial progenitor cell; FN, fibronectin; GelMA, gelatin methacryloyl; HA, hyaluronic acid; HS, heparan sulfate; HSC, hematopoietic stem cell; HUVEC, human umbilical vein endothelial cell; iPSC, induced pluripotent stem cell; MM, multiple myeloma; mPC, monoclonal plasma cell; MSC, mesenchymal stromal cell; nHA, nano-hydroxyapatite; NK, natural killer; NR, not reported; OB, osteoblast; OC, osteoclast; O-MSC, osteogenic mesenchymal stromal cell; PC, plasma cell; PCL, polycaprolactone; PEGDA, polyethylene glycol diacrylate; PGMA, poly(glycerol monomethacrylate); PHPMA, poly(2-hydroxypropyl methacrylate); polyHIPE, polymerized high internal phase emulsion; TME, tumor microenvironment.

Figure 2 complements these general selection criteria by providing a mechanism-oriented framework that links PC-MSC adhesion, pathological bone remodeling, hypoxia, and immune interactions to representative 3D MM model classes and corresponding experimental readouts.

**FIGURE 2:**
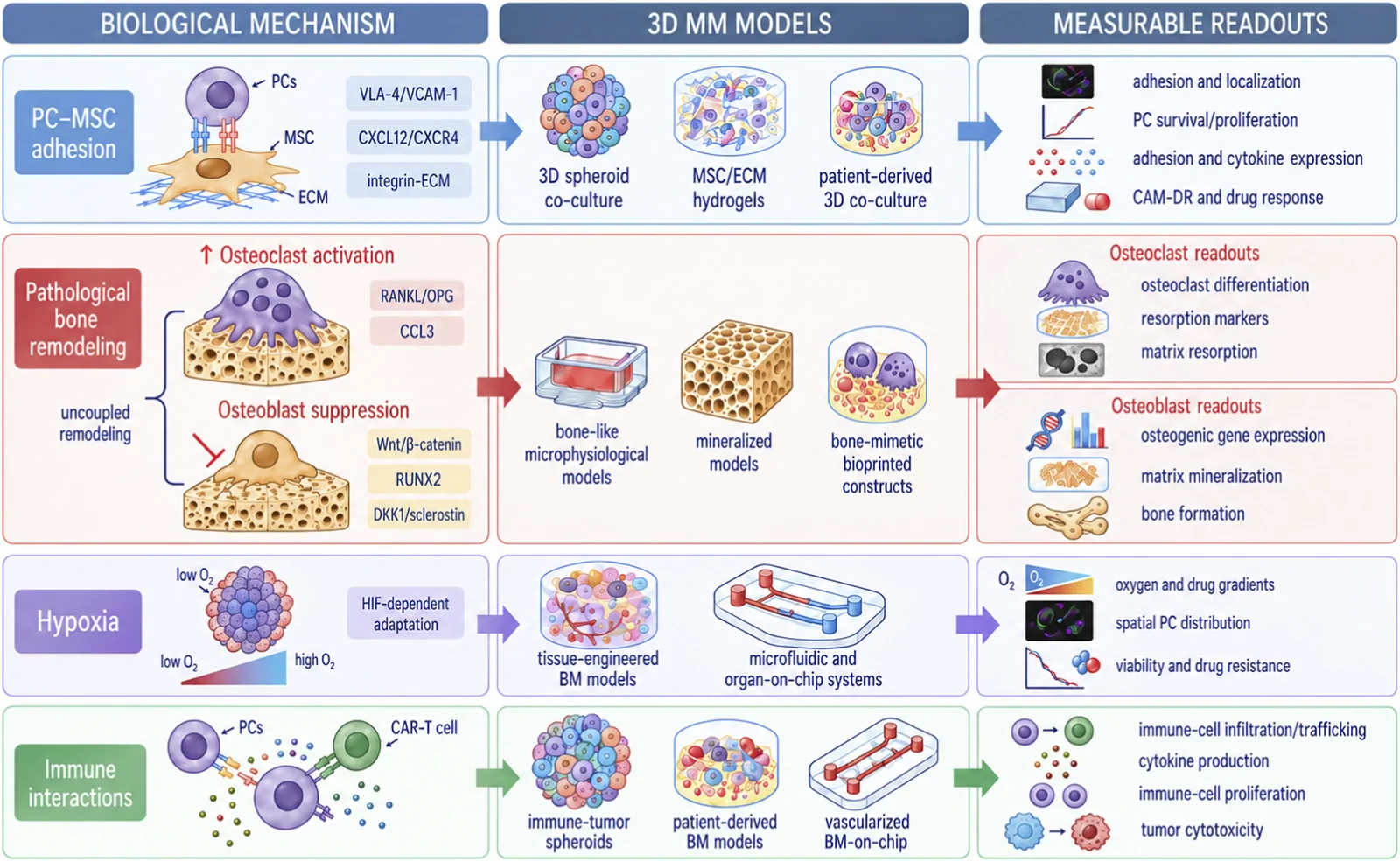
Mechanism-oriented framework linking microenvironmental processes, three-dimensional (3D) multiple myeloma (MM) models, and experimental readouts. The image summarizes four microenvironment-dependent processes relevant to MM (plasma cells (PCs)-mesenchymal stromal cell (MSCs) adhesion, pathological bone remodeling, hypoxia, and immune interactions) and their relationship with representative 3D MM model classes and corresponding experimental readouts. The model-readout associations shown are representative and do not imply that every readout is available on all platforms within a given group.

## 5 Three-dimensional models as platforms for therapeutic target identification

The value of 3D MM models extends beyond their ability to reproduce selected features of the BM niche. By preserving tumor-niche interactions that regulate malignant PC survival and therapeutic response, these platforms can be used to functionally interrogate microenvironment dependent mechanisms of drug resistance and treatment escape (De La Puente et al., 2015; de Jong et al., 2021; Belloni et al., 2018; Alhallak et al., 2021). Table 2 summarizes the main microenvironment dependent therapeutic targets discussed in this section and their current level of evidence in 3D MM models.

**TABLE 2:** Microenvironment dependent therapeutic targets in multiple myeloma (MM) and their level of evidence in three-dimensional (3D) models**.** Targets and pathways are grouped according to their biological function and classified as directly validated in 3D MM models, investigated in 3D MM models but not yet functionally validated as therapeutic targets, or proposed for future 3D validation. Representative 3D platforms and potential applications are reported for each target class.

Target class	Target/pathway	Evidence level in 3D MM models	Representative 3D platform	Potential application
Adhesion and stromal support	VLA-4/VCAM-1, integrins, CXCL12/CXCR4, ECM-dependent survival	Investigated in 3D MM models (Waldschmidt et al., 2022; García-Briega et al., 2025; Jakubikova et al., 2016; Ilic et al., 2024)	MSC-based hydrogels, ECM-containing droplets, microgel co-cultures	Functional interrogation of stromal protection and CAM-DR
Bone remodeling	OB suppression, OC activation, tumor-bone remodeling	Investigated in 3D MM models (Reagan et al., 2014; Visconti et al., 2021)	Bone like microphysiological models, mineralized matrices	Assess pathological uncoupling of bone formation and resorption and identify bone-remodeling vulnerabilities
Bone remodeling	BMP6-associated regulation	Directly validated in 3D MM models (Wang et al., 2025)	Humanized BM like scaffold model	Candidate pathway acting on both tumor and skeletal niche
Hypoxia and spatial gradients	HIF-dependent adaptation, oxygen and drug gradients	Investigated in 3D MM models (De La Puente et al., 2015; Zhang et al., 2014; Ghoshal et al., 2025)	Tissue-engineered BM models, microfluidic and organ-on-chip systems	Study hypoxia-dependent survival and therapeutic resistance under spatially heterogeneous conditions
Hypoxia-associated bone effects	IL-32, miR-210	Proposed for future 3D validation (Zahoor et al., 2017; Saba et al., 2018)	Future bone like or microfluidic systems with controlled oxygen gradients	Determine whether hypoxia contributes directly to functional bone-remodeling uncoupling
Metabolic targets	Glutamate metabolism, EAAT1, NF-κB/NFATc1-mediated osteoclastogenesis	Proposed for future 3D validation (Toscani et al., 2025)	Bone like 3D models with functional OCs and mineralized matrices	Test whether EAAT1/glutamate targeting reduces pathological bone resorption
Soluble factor signaling	IL-6/IL-6R	Directly validated in 3D MM models (Wu et al., 2022)	3D-bioprinted bone-mimetic model	Evaluate cytokine-directed strategies to overcome stromal-mediated drug resistance
Soluble factor signaling	IL-11, HGF and other stromal cytokines	Investigated in 3D MM models (Zdzisińska et al., 2009)	Gelatin-sponge scaffolds, stromal 3D co-cultures	Identify candidate soluble mediators of stromal support and therapeutic resistance

### 5.1 Adhesion and stromal targets

Adhesion-dependent and stromal-mediated mechanisms represent major microenvironment dependent vulnerabilities in MM. In the BM niche, malignant PCs receive adhesive and soluble signals from MSCs and ECM components, promoting tumor retention, survival, and CAM-DR (Damiano et al., 1999; Landowski et al., 2003). These interactions activate pro-survival pathways and reduce sensitivity to anti-myeloma agents, making stromal support one of the clearest examples of niche-mediated therapeutic resistance. Because these processes depend on spatial organization and matrix-mediated signaling, 3D stromal and ECM-containing models provide relevant platforms to test whether disrupting microenvironmental protection can improve therapeutic response (Belloni et al., 2018; Jakubikova et al., 2016).

Different 3D platforms have highlighted complementary aspects of stromal and ECM-mediated resistance. MSC-based hydrogel models showed that niche-like stromal organization is associated with ECM molecule expression, adhesion molecules such as integrins and N-cadherin, and CXCL12 production. This organization supported primary MM cell proliferation and increased CXCR4 expression, indicating that 3D MSC-based systems can capture stromal adhesion and CXCL12/CXCR4-dependent interactions more effectively than simplified cultures (Jakubikova et al., 2016). Collagen-containing droplets further demonstrated that ECM-rich environments can maintain stromal support, preserve CXCL12 expression, and reduce MM cell sensitivity to bortezomib (Ilic et al., 2024). Similarly, microgel-based co-cultures incorporating BM-MSCs and ECM-related components provide a matrix-rich setting to investigate tumor-stroma-matrix interactions and their contribution to drug response (García-Briega et al., 2025).

Patient-derived 3D co-culture systems add a functional *ex vivo* layer by linking stromal support with reduced sensitivity to bortezomib and auranofin in primary samples (Waldschmidt et al., 2022). Together, these studies demonstrate that 3D stromal and ECM-mimetic models can functionally interrogate adhesion-, CXCL12/CXCR4-, and matrix-dependent mechanisms of MM resistance (Waldschmidt et al., 2022; García-Briega et al., 2025; Jakubikova et al., 2016; Ilic et al., 2024).

### 5.2 Bone remodeling targets

Bone remodeling pathways represent a key class of microenvironment dependent targets in MM, because myeloma bone disease is driven by reciprocal interactions between malignant PCs and the skeletal niche. In this context, 3D bone like models provide a functional setting to investigate how MM cells disrupt the balance between OB-mediated bone formation and OC-mediated bone resorption. This is particularly relevant for therapeutic target identification, since effective strategies should ideally limit tumor growth while restoring bone homeostasis and reducing tumor-supportive signals from the skeletal compartment (Reagan et al., 2014; Visconti et al., 2021; Wang et al., 2025).

Early bone like models demonstrated that MM cells can directly suppress osteogenic differentiation and induce niche abnormalities associated with tumor growth, stromal dysfunction, endothelial remodeling, and CAM-DR (Reagan et al., 2014). More specialized microphysiological models extended this approach by recapitulating active tumor-bone remodeling. In these systems, MM cells reduced matrix mineralization, increased OC-associated resorption markers, and suppressed osteogenic gene expression, thereby reproducing central features of myeloma bone disease (Visconti et al., 2021). These platforms therefore provide complementary functional measures of tumor activity, osteogenic dysfunction, and bone resorption that can be used to interrogate bone-remodeling pathways in a MM-specific microenvironment.

Humanized BM like scaffold models further supported the identification of BMP6 as a multifunctional regulator of myeloma bone disease. BMP6 promoted bone formation, reduced osteoclastogenesis, attenuated tumor-supportive stromal features, and directly inhibited MM cell proliferation, suggesting that selected targets may act simultaneously on malignant PCs and the surrounding skeletal niche (Wang et al., 2025). Together, these studies illustrate how 3D bone like systems can be used to assess whether candidate pathways simultaneously regulate malignant PCs and the skeletal compartment (Reagan et al., 2014; Visconti et al., 2021; Wang et al., 2025).

Despite the central role of pathological bone remodeling in MM, only a limited number of 3D models have been specifically designed to reproduce both OB dysfunction and OC activation. Most available platforms assess selected features of bone remodeling, such as impaired osteogenic differentiation, reduced matrix mineralization, or increased OC-associated resorption, whereas only a few studies have directly investigated or pharmacologically validated specific molecular targets within these compartments. Consequently, the capacity of current 3D systems to model the reciprocal coupling between bone formation, bone resorption, and tumor growth remains only partially explored. Further studies are therefore needed to develop standardized multicellular bone like models incorporating functionally mature OBs, OCs, and mineralized matrices, and to determine whether candidate bone-remodeling targets retain their biological and therapeutic relevance under MM-specific microenvironmental conditions.

### 5.3 Hypoxia-associated targets

As MM progresses, cellular and biochemical remodeling of the BM niche creates spatially heterogeneous conditions that influence malignant PC survival and therapeutic response (de Jong et al., 2021; Muz et al., 2014). Within this remodeled microenvironment, local differences in oxygen availability, paracrine signaling, metabolic activity, and drug diffusion can generate adaptive niches that are poorly reproduced in conventional 2D cultures. By partially recreating these gradients, 3D and microfluidic models provide a relevant framework to investigate how hypoxia, metabolic adaptation, and cytokine-mediated signaling contribute to MM cell survival and microenvironment-mediated resistance (De La Puente et al., 2015; Alhallak et al., 2021).

Hypoxia promotes MM cell adaptation through activation of hypoxia-inducible factor (HIF)-dependent pathways. Tissue-engineered 3D BM models have linked spatial organization, oxygen and drug gradients with enhanced MM cell growth and increased resistance to anti-myeloma agents compared with conventional 2D systems (De La Puente et al., 2015). These findings indicate that oxygen availability is not merely an experimental culture parameter but a functional component of the tumor-supportive BM niche. Moreover, hypoxia contributes directly to myeloma bone disease by modifying tumor-derived signals that regulate OC and OB activity. One of the best characterized mechanism involves the HIF-1α-dependent induction of IL-32 in MM cells. Increased IL-32 expression has been associated with skeletal involvement and bone loss in MM (Zahoor et al., 2017). Moreover, IL-32 released by hypoxic MM cells, including IL-32 transported through extracellular vesicles, promotes OC differentiation *in vitro* and *in vivo*, providing a mechanistic connection between local oxygen deprivation, tumor-derived signaling, and pathological bone resorption (Zahoor et al., 2017).

At the same time, hypoxia may contribute to defective bone formation by altering the communication between MM cells and osteogenic progenitors. HIF-1α-dependent regulation of miR-210 modifies the expression of adhesion molecules and soluble mediators involved in tumor-stromal interactions, including VLA-4, CXCR4, IL-6, and TGF-β (Saba et al., 2018). Microvesicles released by hypoxic MM cells have also been reported to impair the osteogenic differentiation of BM-MSCs *in vitro* (Saba et al., 2018). Although this mechanism requires further confirmation in more complex experimental systems, it suggests that hypoxia may reinforce the pathological uncoupling of bone remodeling by simultaneously promoting osteoclastogenic signaling and suppressing OB differentiation.

Despite this evidence, hypoxia has been investigated in 3D MM models primarily in relation to malignant PC survival, spatial adaptation, and drug resistance. Its contribution to the functional uncoupling of bone resorption and formation remains poorly characterized, as few platforms integrate controlled oxygen gradients with active bone remodeling. Bone like microfluidic models integrating controlled oxygen gradients with functional bone-remodeling measures will therefore be important for determining whether hypoxia associated pathways represent tractable microenvironment dependent targets in MM.

### 5.4 Metabolic and soluble factor targets

Beyond hypoxia-dependent mechanisms, additional metabolic and soluble factor alterations within the MM BM niche may provide distinct microenvironment dependent therapeutic targets. Glutamate accumulation in the BM of patients with MM promotes OC formation and bone resorption through EAAT1-mediated activation of NF-κB- and NFATc1-related signaling (Toscani et al., 2025). Although this mechanism has not yet been extensively investigated in bone like 3D MM models, platforms incorporating functional OCs and mineralized matrices could be used to determine whether inhibition of glutamate transport reduces pathological bone resorption under tumor-supportive conditions.

Cytokine and growth-factor networks also contribute to MM cell survival, stromal protection, and therapeutic resistance. Gelatin-sponge scaffolds and 3D-bioprinted MM models have shown that spatial stromal organization can modify the production of IL-11, HGF, soluble IL-6 receptor, and IL-6-related signals, thereby influencing MM cell behavior and response to bortezomib (Wu et al., 2022; Zdzisińska et al., 2009). In a bioprinted bone-mimetic model, increased IL-6 production was associated with stromal-mediated resistance to bortezomib, whereas inhibition of IL-6 signaling with tocilizumab restored drug sensitivity (Wu et al., 2022).

Overall, these studies identify metabolic and cytokine-mediated pathways as candidate microenvironment dependent targets, although direct functional validation in advanced 3D MM models remains limited. Multicellular bone like platforms may therefore be particularly useful for determining whether these pathways simultaneously regulate MM cell survival and pathological bone remodeling.

## 6 Computational and artificial intelligence-based approaches for modeling the multiple myeloma bone microenvironment

AI and computational approaches are increasingly used in MM to integrate complex imaging, spatial, molecular, and clinical data (Romero et al., 2024). However, their direct application to experimental 3D MM models remains relatively limited. Most AI-based studies in MM currently focus on imaging, histopathology, spatial profiling, or multimodal clinical datasets rather than on the direct analysis of 3D culture systems (Romero et al., 2024). Nevertheless, these approaches may provide important information for the design and interpretation of 3D models by identifying spatial and molecular features of the BM microenvironment that should be reproduced experimentally.

Recent studies illustrate the potential of AI to characterize the MM microenvironment directly in patient samples. Hagos et al. developed deep-learning pipelines for multiplex immunohistochemistry images of BM trephine biopsies, allowing spatial mapping of malignant PCs in relation to immune, stromal, and bone-associated compartments (Hagos et al., 2024). Their analysis showed that spatial heterogeneity, rather than cell density alone, distinguished MGUS from newly diagnosed MM and demonstrated substantial remodeling of the BM microenvironment following treatment (Hagos et al., 2024). Such approaches provide tissue-level spatial information that may serve as a reference for evaluating whether engineered 3D models reproduce relevant features of the native MM niche. Similarly, spatial transcriptomic and single-cell RNAseq studies can identify disease-associated cellular populations and spatially restricted microenvironmental states, helping to prioritize which components should be incorporated into BM like, bone like, patient-derived, or organ-on-chip systems. Yip et al. identified spatially defined malignant PC subpopulations across healthy, premalignant, and active MM samples (Yip et al., 2025), while large-scale single-cell datasets have revealed patient-specific immune and hematopoietic programs associated with disease progression and clinical outcome (Pilcher et al., 2026).

Computational modeling provides an additional framework for investigating dynamic tumor-microenvironment interactions that are difficult to capture experimentally. Bishop et al. developed a spatiotemporal hybrid agent-based model of the MM-BME and showed that both intrinsic PC properties and BME-mediated drug resistance contribute to the emergence and heterogeneity of treatment-resistant clones (Bishop et al., 2024). Integrating such computational predictions with experimental 3D systems may help identify niche-dependent variables for functional validation and guide the development of more focused bone like models.

The direct application of AI to 3D MM platforms remains limited; however, studies in different cancer models provide proof of concept for its potential use. For example, in a 3D microfluidic bladder cancer model, tumor cells with different levels of gemcitabine resistance were co-cultured with endothelial cells and analyzed using a convolutional neural network (Tak et al., 2024). The algorithm was able to distinguish different levels of drug resistance from image-derived features, demonstrating how AI can convert complex phenotypic information generated by a 3D model into quantitative measures of therapeutic response (Tak et al., 2024). Similarly, deep-learning approaches have been used in tumor spheroids and patient-derived organoids to automate segmentation, quantify morphology and growth, and monitor treatment-induced changes (Liu et al., 2024). Similar strategies could be adapted to MM-specific BM like and bone like platforms to quantify malignant PC spatial organization, tumor-stroma interactions, immune-cell infiltration, matrix remodeling, and heterogeneous drug responses. From an analytical perspective, however, the application of AI to 3D MM models is challenged by the limited size of available datasets, susceptibility to batch-related variability, and the risk of overfitting (Homeyer et al., 2022; Asif et al., 2023). Independent validation will therefore be necessary to determine whether AI-derived features are reproducible across different datasets and model systems (Homeyer et al., 2022). Thus, rather than representing an established component of current 3D MM modeling, AI should presently be viewed as an emerging complementary tool that may improve the design, quantitative analysis, and interpretation of increasingly complex experimental platforms.

## 7 Translational applications, limitations and future perspectives

The potential translational value of 3D MM models derives from their ability to assess therapeutic responses in human-based BM like settings that incorporate selected features of the native BM niche more effectively than conventional 2D cultures (De La Puente et al., 2015; Belloni et al., 2018; Alhallak et al., 2021). Rather than providing only a measure of intrinsic tumor-cell sensitivity, these platforms can reveal how the surrounding niche influences drug response, resistance, and treatment escape (De La Puente et al., 2015; Belloni et al., 2018). One promising translational application is the use of patient-derived 3D cultures as functional platforms for *ex vivo* drug testing (Alhallak et al., 2021). These systems may preserve part of the heterogeneity of individual BM samples and allow therapeutic response to be assessed in a context that better reflects patient-specific tumor-niche interactions and disease stage (Alhallak et al., 2021; Waldschmidt et al., 2022). In this sense, 3D models could complement molecular profiling by adding a functional layer of information. This may be particularly useful in relapsed or refractory MM, where clonal evolution, prior treatment exposure, and microenvironment-mediated resistance often make therapeutic selection difficult (Rafae et al., 2024; Bishop et al., 2024). If integrated with genomic, transcriptomic, spatial, and clinical data, patient-derived 3D assays may contribute to functional precision medicine by helping to identify candidate patient-specific vulnerabilities and rational drug combinations. However, their ability to predict clinical response at the individual-patient level remains to be established prospectively in larger and independent cohorts (Alhallak et al., 2021).

Importantly, the translational potential of patient-derived 3D models extends beyond direct drug sensitivity testing to the evaluation of immunotherapeutic strategies. Incorporating immune effector cells into a BM like 3D environment could enable investigation of tumor immune interactions, immune-cell infiltration and activation, and mechanisms of resistance to immune-based treatments. Such approaches may be particularly relevant for evaluating therapies whose activity depends on interactions between malignant PCs, immune cells, stromal populations, and ECM. However, the incorporation of primary patient-derived PCs and immune components also introduces important technical challenges. Primary malignant PCs are highly dependent on the BM microenvironment for their survival and function and may show limited viability and phenotypic stability following *ex vivo* isolation. Reconstructing appropriate cellular and matrix interactions may therefore be necessary to extend their maintenance and preserve disease relevant features. At the same time, prolonged culture periods may increase model complexity and variability, whereas short experimental windows may limit the ability to assess delayed therapeutic responses or dynamic changes in the tumor microenvironment. Defining reproducible assay durations that maintain cellular viability and biological relevance while remaining compatible with drug and immunotherapy testing will therefore be an important step toward translational implementation.

Beyond *ex vivo* drug testing, 3D MM models may provide an intermediate preclinical setting for validating microenvironment dependent therapeutic targets and combination strategies, as discussed in Section 5 (Belloni et al., 2018; Alhallak et al., 2021; Visconti et al., 2021; Wang et al., 2025). Despite this potential, several limitations still restrict the use of 3D MM models (Bottaro et al., 2025; Ybarra et al., 2025). Most current systems reproduce only selected aspects of the BM niche, and increasing complexity does not necessarily make a model more informative (Bottaro et al., 2025; Ybarra et al., 2025; Belloni et al., 2018). The most informative platform is therefore the one that best captures the microenvironmental mechanism relevant to the question being addressed. Standardization also remains a major challenge, because differences in model design, culture conditions, and analytical readouts can substantially influence cell behavior and therapeutic response (Bottaro et al., 2025; Ybarra et al., 2025; Ilic et al., 2024). In patient-derived systems, additional variability derives from sample quality, tumor burden, cellular composition, and the limited viability of primary malignant PCs *ex vivo* (Alhallak et al., 2021; Waldschmidt et al., 2022). Although this heterogeneity complicates experimental standardization, it may also represent an important biological feature of patient-derived models, allowing inter-patient differences in therapeutic response and treatment resistance to be investigated.

Importantly, biological relevance should not be equated with clinical predictivity. Most 3D MM models have been validated primarily against experimental or molecular endpoints, whereas direct comparisons between *ex vivo* drug responses and clinical outcomes remain uncommon and have generally involved limited patient cohorts. Although some patient-derived platforms have shown encouraging correlations with clinical response, these findings require prospective validation in larger and independent patient populations before such assays can be considered clinically predictive.

Future progress will therefore depend on modular and standardized platforms. Rather than aiming to reproduce the entire BM microenvironment in a single system, future models should be designed around defined biological or therapeutic questions. For example, a model designed to study myeloma bone disease should reproduce different features than one designed to assess immune-cell infiltration or hypoxia-driven resistance. This modular strategy could make 3D systems more reproducible, easier to compare, and more informative for translational research (Bottaro et al., 2025; Ybarra et al., 2025; Belloni et al., 2018). Integration with patient-derived data and clinically annotated datasets may further improve model interpretation and provide a framework for establishing predictive performance. Advanced imaging and AI-based approaches, discussed in the previous section, may facilitate this process by enabling quantitative analysis of increasingly complex experimental outputs (Hagos et al., 2024; Yip et al., 2025; Bishop et al., 2024; Pilcher et al., 2026; Diosdi et al., 2025). Therefore, the next step will be to determine whether biologically informative 3D MM models can be developed into standardized platforms with clinically validated performance for drug testing and therapeutic stratification.

## 8 Conclusion

3D MM models provide complementary experimental platforms for investigating tumor-microenvironment interactions that are difficult to reproduce in conventional 2D systems. Their value should be assessed according to their ability to reproduce defined biological mechanisms through appropriate cellular, matrix, and functional components, rather than according to increasing structural complexity. Distinguishing general BM like systems from models incorporating functional bone associated features will be particularly important for investigating myeloma bone disease. Future development should focus on defining which biological features are essential for each application and on establishing whether these features translate into reproducible and functionally meaningful outputs. Integration with computational and AI-assisted approaches may further support the refinement and interpretation of these models. Rather than replacing established preclinical approaches, 3D MM models are likely to contribute as complementary systems for mechanistic investigation and translational research.
